# Annual incidence and prevalence of injuries in elite male academy cricketers: A 4-year prospective cohort study

**DOI:** 10.1016/j.jsampl.2023.100050

**Published:** 2023-12-26

**Authors:** Amy Williams, Nicholas Peirce, Steve Griffin, Ben Langley, Carly McKay, Keith A. Stokes, Sean Williams

**Affiliations:** a Centre for Health, and Injury & Illness Prevention in Sport, Department of Health, University of Bath, United Kingdom; b UK Collaborating Centre on Injury & Illness Prevention in Sport (UKCCIIS), Bath and Edinburgh, United Kingdom; c England and Wales Cricket Board, National Cricket Performance Centre, Loughborough University, United Kingdom; d Mumbai Indians, Mumbai, India

**Keywords:** Epidemiology, Cricket sport, Sport medicine, Adolescent, Injury surveillance, Injury prevention

## Abstract

**Objectives:**

The aim of this study was to describe the epidemiology of injuries; time-loss and non-time loss, in elite male academy cricket.

**Design:**

Prospective cohort analysis.

**Methods:**

Annual injury incidence and prevalence from all cricket related injuries were calculated for 348 male academy players (under-13 to under-18) from the 18 First-Class County Cricket clubs in England and Wales across four years (2017/18, 2018/19, 2020/21 and 2021/22), in accordance with the updated consensus statement for injury surveillance methods in cricket.

**Results:**

The average annual injury incidence was 115.0 injuries/100 players/year, with similar rates between time-loss (59.7 injuries/100 players/year) and non-time loss injury incidence (55.3 injuries/100 players/year). On average, 8.5 ​% of players were unavailable on any given day of the year due to injury. Match injury incidence (48.8 injuries/100 players/year) was higher than cricket-based training (25.2 injuries/100 players/year), gym-based training, illness, and ‘other’ injury incidences. Match bowling was the activity associated with the highest total (17.7 injuries/100 players/year), time-loss (10.3 injuries/100 players/year) and non-time loss (7.4 injuries/100 players/year) injury incidence. The lumbar spine was the body location most frequently injured (15.3 injuries/100 players/year) and was the most prevalent body location injured (2.9 ​% of players).

**Conclusions:**

The findings from this study provide, robust evidence of the extent of the injury problem in elite male academy cricketers. Bowling poses the greatest risk to players and the lumbar spine is the most common and prevalent injury location.

## 1 Introduction

The first stage in van Mechelen's injury prevention model is establishing the extent of the current problem through injury surveillance [1]. Surveillance data allows for the identification of injury risk factors [1] and longitudinal studies with similar methodologies and measures allow comparisons between different settings. In 2016, an updated international consensus statement was published with methods of injury surveillance in cricket, to improve the quality and consistency of the research [2]. Since then, there have been numerous longitudinal epidemiological studies describing the injury risk in elite male cricket from Australia [3], England [4], and New Zealand [5]. Consistent findings show hamstring injuries to be the most common injury incidence, lumbar spine injuries to be the most prevalent and bowling to pose the highest risk of injury. However, there is a paucity of data on injuries in elite male academy players who have the potential to play first-class cricket in the literature.

There are a few studies that have reported time-loss injury epidemiology in academy cricketers in the literature. In the Asian Cricket Council Under-19 Elite Cup an overall injury incidence of 292/10 ​000 player hours was observed, with the highest proportion (50 ​%) of injuries reported to the lower limb and associated with fielding (46 ​%) [6]. In South African provincial under-15, under-17 and under-18 cricket team's, injuries were also predominately to the lower limb (38 ​%) whilst bowling carried the greatest risk of injury (44 ​%) [7]. More recently, similar findings have been described for young professional academy cricketers in Lahore, where again lower limb injuries were most common (42 ​%) and injuries occurred equally between bowling and fielding (41 ​%) [8]. However, the inconsistent methodologies limit the extent to which these studies can be compared. Despite the updated consensus aiming to improve the quality of injury surveillance reporting in cricket, there is a lack of research in elite academy cricket utilising these recommendations. High-quality surveillance studies with consistent methodologies are paramount to understanding the injury situation in male academy cricketers, and ultimately provide the foundations for developing injury prevention strategies.

The inclusion of non-time loss injuries in epidemiology studies can develop a greater understanding of the burden of injury and locations of physical stress on athletes [9] and inform the care that medical staff provide to academy cricketers. In the academy setting, growth-related injuries may also be prevalent and are best understood using non-time loss definitions as they are often transient and gradual onset in nature [10]. Consequently, the reporting of non-time loss injuries could aid with the development of injury prevention strategies aimed at this elite academy setting.

Understanding the injury risk in elite male academy cricketers is important as injuries in young athletes are pernicious to long term physical health [11], mental health [11], player progression and obtaining professional contracts [12], and lead to drop out of sport [11]. Furthermore, there is no alignment in match scheduling and game formats between academy and professional cricketers, with the majority of academy games played over one-day, making it difficult to translate injury prevention strategies from professional to academy cricket. Similarly, it is important to not generalise the rate of injury from school and community cricket players to elite academy cricket players as playing standards and skill proficiency will differ, along with the number of training and match days, potentially influencing the rate of injury. Accordingly, the aim of this study was to describe the epidemiology of injuries (both time-loss and non-time loss) in elite male academy cricket in England and Wales.

## 2 Methods

This prospective cohort study included all registered male academy players from the 18 First Class County Cricket (FCCC) clubs in England and Wales as part of the England and Wales Cricket Board (ECB) injury surveillance programme from 1st October 2017 to 30th September 2022. The FCCC academies in England and Wales consists of the Academy Programme where players are registered to a county academy, aged under-13 to under-18 and deemed to have the potential to play FCCC. As such the number of players a FCCC club registers is not uniform and varies year to year. All players (and parent/carer for players under-18) provided written, informed consent for the use of their data for research purposes by the ECB and University research partner at the time of annual registration. Ethical approval was obtained from the University of Bath, Research Ethics Approval Committee for Health (REACH reference number: EP 20/21 065) and was carried out in accordance with The Code for Ethics of the World Medical Association (Declaration of Helsinki) for experiments involving humans. Participant information sheets were sent to all 18 FCCC clubs academy medical staff to distribute to players to inform them of the study and provide opportunity to withdraw from the study.

All injuries were recorded by medical staff (physiotherapist or doctor) at each FCCC club using a purpose-built online database (Cricket Squad [The Sports Office, UK]) at the time of injury. In line with the updated consensus statement for methods of injury surveillance in cricket [2], a medical attention injury definition was utilised whereby a health-related condition that required medical (or medical staff) attention and had the potential to affect cricket training or playing, including time-loss and non-time loss injuries. An injury was considered a time-loss injury if it resulted in a player being considered unavailable for match-play, irrespective of whether a match or training was actually scheduled on that day [2]. The definition of injury, in line with the updated consensus statement for injury surveillance methods, is inclusive of illness [2]. All injuries logged by medical staff also included details on the body region injured and diagnosis coded from the Orchard Sport Injury Classification System (OSICS) Version 10 [13], problem type (match, cricket-based training, gym-based training, illness, other [e.g. travel related]) and activity at time of injury (batting [match/training], bowling [match/training], fielding [match/training], wicket keeping [match/training], gym-based training). Lumbar spine injuries were further explored to identify specific injury diagnosis and activity at time of injury due to the high incidence and prevalence rates observed.

Prior to data sharing with the University research partner, a gatekeeper at the ECB (physiotherapist) extracted all relevant data from ‘Cricket Squad’ where players were deidentified and names replaced with a unique numerical ID. The lead author checked the dataset for errors such as duplicates of injury entries, body regions that did not match to the OSICS code, and records that remained open, were incomplete or required updating. Where necessary, the gatekeeper at the ECB corrected errors and removed duplicates.

It was not possible to obtain the number of match days played in this cohort of players over the study period due to the varying competitions undertaken by players in each academy. Therefore, to adhere to the updated consensus statement, annual injury incidences were calculated with the unit of per 100 players per year. The number of academy players was taken from formal registration numbers. In line with the domestic FCCC season, a year was deemed to run from October 1 to September 30. The 2019/20 year was excluded from this study due to the impact of COVID-19, which severely limited cricket played in this cohort. Accordingly, annual total, time-loss and non-time loss injury incidences were calculated from the number of new and recurrent injuries in all cricket related activities, inclusive of gradual and insidious onset using the following formula:

Injury incidence = (no. of injuries / no. of players) × 100

Annual general time-loss injury prevalence was calculated for time-loss injuries as the percentage of players unavailable on any given day of the year:

Injury prevalence = (no. of days lost due to injury / (no. players × no. days)) ×100

Injury incidence and prevalence were summarised with mean and 95 ​% confidence intervals (CI) using the Poisson and Exact Binomial method, respectively [14].

## 3 Results

There were on average, 182 (±24.0) academy players registered each year across the 18 academies. Over the four years, a total of 836 injuries (434 time-loss and 402 non-time loss) were recorded in 348 players, with an average of 209 (±18.2) injuries recorded each year.

The overall injury incidence over the four years was 115.0 injuries/100 players/year (95 ​% CI 107.5, 123.1), with 59.7 (95 ​% CI 54.3, 65.6) time-loss and 55.3 (95 ​% CI 50.1, 61.0) non-time loss injuries/100 players/year observed (Table 1). There was a decrease in annual non-time loss injury incidence in 2020/21 compared to the first two years and in 2021/22 compared to 2017/18. There was a similar rate of total and time-loss annual injury incidence between the years. Injury prevalence on average was 8.5 ​% (95 ​% CI 6.1 ​%, 10.0 ​%), with rates similar between each year (Table 1).

**Table 1:** Annual injury incidence (per 100 players per year) and prevalence (percentage of players unavailable on any given day) from 2017/18 to 2021/22, with 95 ​% CI.

Year	Injury incidence			Injury prevalence
	Total	Time-loss	Non-time loss	
2017/18	123.2 (107.3, 141.4)	49.4 (39.7, 61.4)	73.8 (61.7, 88.2)	8.9 ​% (4.8 ​%, 13.4 ​%)
2018/19	126.4 (110.2, 144.9)	60.7 (49.9, 74.0)	65.6 (54.3, 79.3)	14.4 ​% (8.0 ​%, 18.0 ​%)
2020/21	109.8 (96.6, 124.8)	69.2 (58.9, 81.2)	40.7 (32.9, 50.2)	6.6 ​% (3.4 ​%, 10.1 ​%)
2021/22	103.8 (90.1, 119.5)	57.0 (47.1, 68.9)	46.8 (37.9, 57.7)	5.3 ​% (2.5 ​%, 9.2 ​%)
Overall	115.0 (107.5, 123.1)	59.7 (54.3, 65.6)	55.3 (50.1, 61.0)	8.5 ​% (6.1 ​%, 10.0 ​%)

Match total, non-time loss and time-loss annual injury incidence (48.8 injuries/100 players/year, 95 ​% CI 44.0, 54.2; 25.7 injuries/100 players/year, 95 ​% CI 22.3, 29.7; 23.1 injuries/100 players/year, 95 ​% CI 19.9, 26.9, respectively) was higher than cricket-based training, gym-based training, illness and ‘other’ (Fig. S1A). Annual match injury prevalence was 4.2 ​% (95 ​% CI 2.8 ​%, 5.8 ​%), cricket-based training 2.0 ​% (95 ​% CI 1.1 ​%, 3.3 ​%), gym-based training 0.3 ​% (95 ​% CI 0.0 ​%, 1.0 ​%), ‘other’ 0.9 ​% (95 ​% CI 0.4 ​%, 2.0 ​%) and illness 0.4 ​% (95 ​% CI 0.1 ​%, 1.2 ​%) (Fig. S1B).

**Fig. 1:**
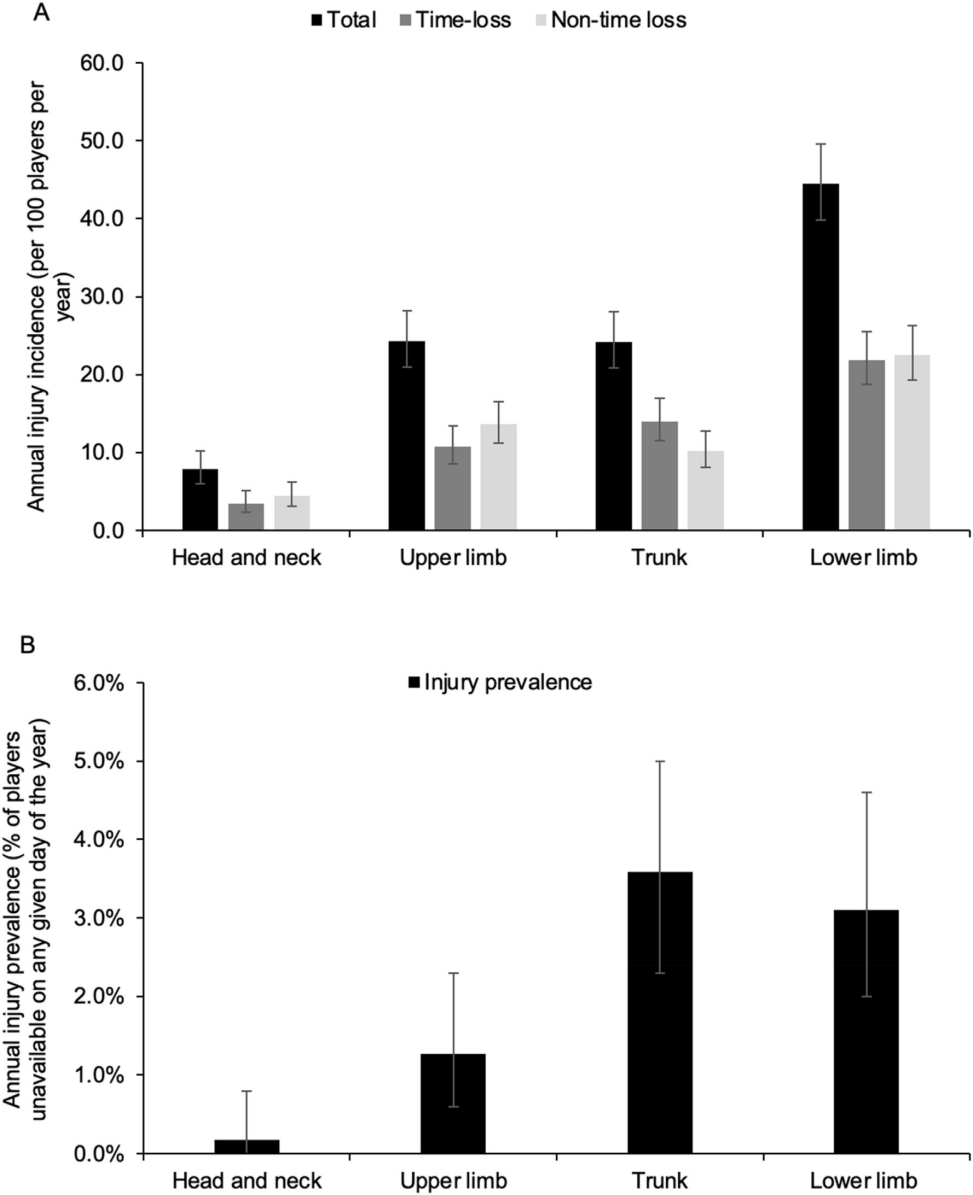
A) The average annual injury incidence (per 100 players per year) and B) the average annual injury prevalence (percentage of players unavailable on any given day of the year) by body region, with 95 ​% CI error bars.

The lower limb was the most common body region injured (44.4 injuries/100 players/year, 95 ​% CI 39.8, 49.5) and was higher than upper limb (24.3 injuries/100 players/year, 95 ​% CI 21.0, 28.2), trunk (24.2 injuries/100 players/year, 95 ​% CI 20.9, 28.1) and head and neck injuries (7.8 injuries/100 players/year, 95 ​% CI 6.0, 10.2) (Fig. 1A). The trunk (3.6 ​%, 95 ​% CI 2.3 ​%, 5.0 ​%), lower limb (3.1 ​%, 95 ​% CI 2.0 ​%, 4.6 ​%) and upper limb (1.3 ​%, 95 ​% CI 0.6 ​%, 2.3 ​%) injury prevalence was higher than the head and neck region (0.2 ​%, 95 ​% CI 0.0 ​%, 0.8 ​%) (Fig. 1B). Injuries to the lumbar spine were the highest total (15.3 injuries/100 players/year, 95 ​% CI 12.7, 18.4), time-loss (8.4 injuries/100 players/year, 95 ​% CI 6.5, 10.8) and non-time loss (6.9 injuries/100 players/year, 95 ​% CI 5.2, 9.1) annual injury incidence by body location. Lumbar spine injury prevalence (2.9 ​%, 95 ​% CI 1.7 ​%, 4.3 ​%) was higher than all other body locations but similar to the knee (1.0 ​%, 95 ​% CI 0.4 ​%, 2.0 ​%) and ankle (0.8 ​%, 95 ​% CI 0.3 ​%, 1.8 ​%) (Table S1).

Upon further investigation into lumbar spine injuries due to the high injury incidence and prevalence, 36.1 ​% of the time-loss lumbar spine injuries were stress fractures, equating to an annual injury incidence rate of 3.0 time-loss lumbar spine stress fractures/100 players/year (95 ​% CI 2.8, 3.3). Lumbar spine injuries mostly occurred from bowling (53.2 ​%) (59.0 ​% of time-loss lumbar spine injuries). Further details of the type of illnesses recorded each year are detailed in Table S2. COVID-19 illness and related isolations accounted for 79.5 ​% and 52.5 ​% of all illnesses in 2020/21 and 2021/22, respectively.

**Fig. 2:**
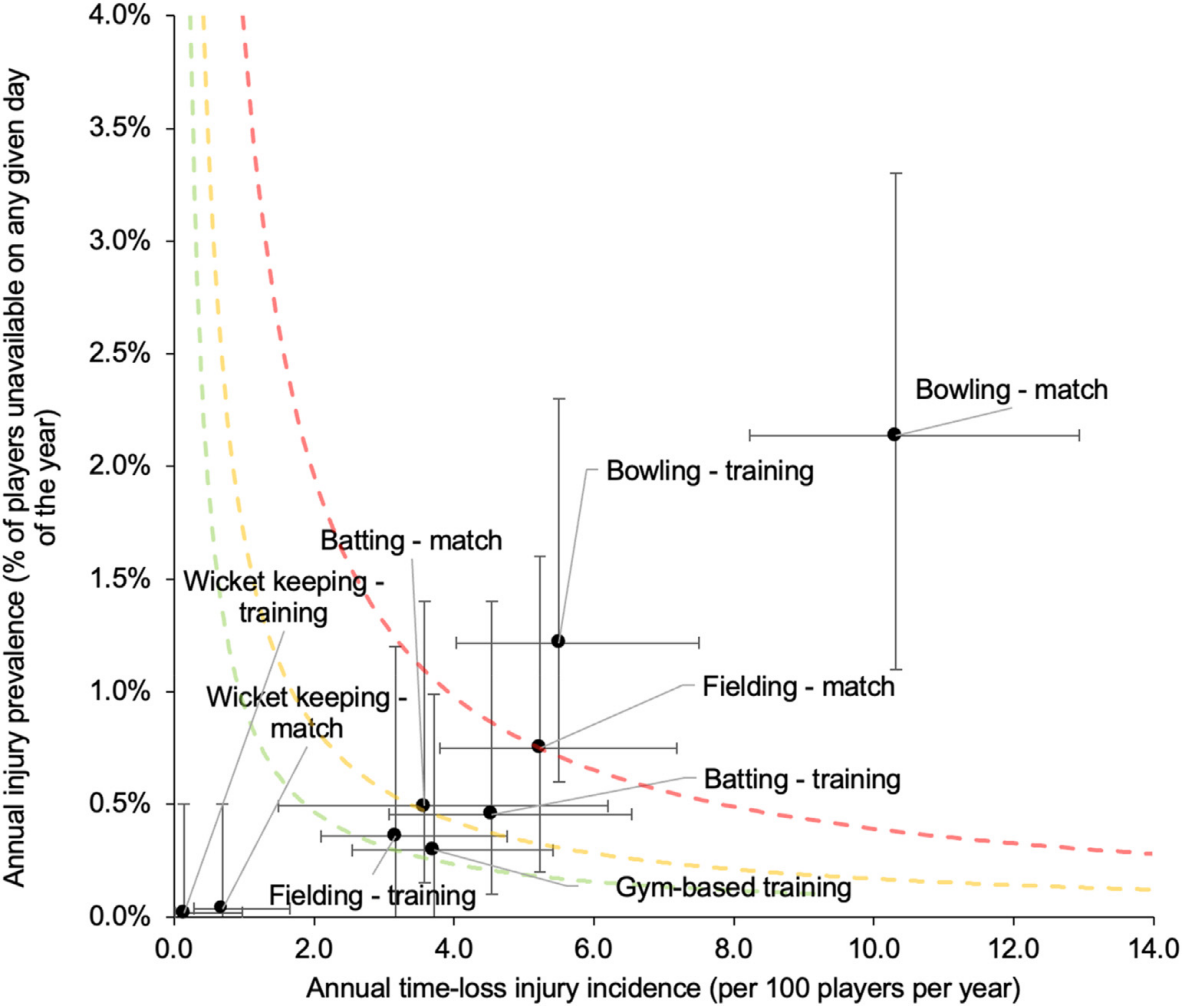
The average annual time-loss injury incidence and annual injury prevalence for each activity at the time of injury over the four years, with 95% CI error bars. Green line, values to the left and below represent under the 25th burden percentile, low risk injuries. Orange line, values to the left and below represent the 50th burden percentile, low-medium risk injuries. Red line, values to the left and below represent the 75th burden percentile, medium–high risk injuries. Values above and to the right of the red line are most-high risk injuries [30].

Match bowling was the activity associated with the highest total and time-loss injury incidence (17.7 injuries/100 players/year, 95 ​% CI 14.9, 21.1) and was higher than all other match and training-based activities (Table S3). Similarly, the highest annual injury prevalence was associated with match bowling (2.1 ​%, 95 ​% CI 1.1 ​%, 3.3 ​%), followed by training bowling (1.2 ​%, 95 ​% CI 0.6 ​%, 2.3 ​%), match fielding (0.7 ​%, 95 ​% CI 0.2 ​%, 1.6 ​%), match batting (0.5 ​%, 95 ​% CI 0.2 ​%, 1.4 ​%) and training batting (0.5 ​%, 95 ​% CI 0.1 ​%, 1.4 ​%). Figure 2 shows match bowling to be the most burdensome activity associated with injury in terms of time-loss injury incidence and injury prevalence.

There was a similar average time-loss (59.7 injuries/100 players/year, 95 ​% CI 54.3, 65.6) and non-time loss (55.3 injuries/100 players/year, 95 ​% CI 50.1, 61.0) injury incidence over the four years. Similarly, the distribution of time-loss and non-time loss injury incidence by the body location or activity at the time of injury was similar. There was a similar time-loss and non-time loss injury incidence by problem type, except for illness, where time-loss illnesses (9.6 injuries/100 players/year, 95 ​% CI 7.6, 12.2) were more frequent than non-time loss illnesses (4.8 injuries/100 players/year, 95 ​% CI 3.5, 6.7).

## 4 Discussion

This is the first prospective cohort study of injuries sustained in the elite male cricket academy setting. The results indicate that on average, an elite male academy player can expect approximately one injury every year. In the cohort of academy players, approximately one time-loss and one non-time loss injury will occur for every two players per year. Over the four years, on average 8.5 ​% of players were unavailable for match selection or training on any given day of the year due to injury. Match bowling was the activity associated with the highest total injury incidence and injury prevalence and was deemed the most burdensome activity for injury. Lumbar spine injuries were the most common specific body location injured and caused players to be out of cricket for the longest period of time.

The injury incidence measure used in this study conforms with the updated cricket consensus statement [2]. Previous studies within the male cricket academy population have utilised various incidence rates and units including match-player-days, player-hours, and proportions of total injuries [6,7,15], and as a result, direct comparisons of incidence rates are limited. Comparisons can be made, however, to studies in professional male cricket populations. A similar seasonal time-loss injury incidence of 53.8 injuries/100 players/year was reported in male First-Class English cricket [4]. However, seasonal rather than annual incidence was reported, which would inherently miss injuries sustained outside of the domestic season and pre-season that were captured in this study. Therefore, it is likely that the injury incidence rate in this study is less than what would be reported in male First-Class English cricket when evaluating injuries annually rather than seasonally.

A lower match time-loss incidence was observed in this study (25.7 injuries/100 players/year) compared to First-Class Australian cricket (64.0 injuries/100 players/year) [3]. Professional players will be exposed to more games than academy players, particularly the number of First-Class games, leading these players to be exposed to more potentially injurious events. Furthermore, this study also included the international team that typically plays matches all-year round in comparison to the six-month domestic season, leading to greater exposure to potentially injurious events and a higher risk of sustaining a time-loss injury within a match. Altogether, the incidence of injury in elite academy players in this study appears to be lower than professional male cricket; however, future studies are required to confirm this.

The present study highlighted that match bowling was the activity associated with the highest incidence and prevalence of injury, which is consistent with findings in professional male cricket [[3], [4], [5]]. This has also been noted in South African provincial age group cricket teams, where 44 ​% of all injuries were due to bowling [7]. Bowling injuries are most often associated with fast bowling where evidence from youth and First-Class cricket shows a higher injury incidence and prevalence in fast bowlers compared to spin bowlers, primarily due to the high rate of lumbar spine injuries suffered by fast bowlers [3,16]. In contrast to the present study, findings from Sri Lankan under-15 and under-17 schoolboy cricket and the Asian Cricket Council under-19 Elite Cup, have shown the majority of injuries to occur whilst fielding [6,15]. A high proportion of the fielding injuries in these studies were due to mis-fielding, including catching and throwing, which may indicate a disparity in skill level amongst these players. The players in the present study were part of an elite academy setting and are possibly more skilled in fielding techniques, such as catching, throwing, and retrieving the ball, and thus more protected from fielding injuries. Nevertheless, injury surveillance in male academy cricket is still in its infancy and warrants future research to fully understand the activities with the highest injury risk.

The highest amount of time-loss due to lumbar spine injuries is a consistent finding in professional male cricket [[3], [4], [5]]. However, the finding of the highest incidence of injury in the academy population is particularly concerning given the time-loss from cricket associated with this injury. Lumbar spine injuries most commonly occurred during bowling in this study, consistent with previous research [3]. A high percentage of lumbar spine injuries were identified as lumbar spine stress fractures. Management of these types of stress fracture typically requires four to six months off bowling to allow for complete bone healing with an average of eight months until return to play, attributing to the high prevalence seen in this study [17,18]. Previous research has shown an increased risk of lumbar spine injuries in fast bowlers <22-years-old in First-Class English and Australian cricketers [19,20]. The increased risk in younger players seen in this study could related to the later maturation and peaking of the lumbar spine bone mineral density (BMD) and content (BMC) compared to other skeletal sites, with lumbar spine peak bone mass potentially not attained until mid-twenties [21]. In English fast bowlers the highest lumbar spine bone mineral was observed between 20 and 24-years-old and compared to athletes of other sports had a greater amount of lumbar BMD and BMC [22]. Furthermore, the higher lumbar bone mineral adaptation seen in fast bowlers has been attributed to internal and external loading of the sport over time, thus younger fast bowlers’ lumbar spine may not be equipped to sustain prolonged periods of bowling [22].

The workload of fast bowlers has also been shown to be a significant risk factor, whereby First-Class bowlers who bowled more than 234 deliveries in a 7-day period were more likely to sustain a lumbar stress fracture compared to those who bowled fewer than 197 deliveries [19]. Junior bowler who had less than 3.5 rest days between bowling sessions have also been shown to have an increased risk of injury compared to those who had greater than 3.5 rest days [23]. Although not captured in this study, an increase in workload, through frequency, duration and intensity could be feasible as the older players progress towards playing First-Class cricket. This has been observed in a group of adolescent fast bowlers (14-18-years-old) where all lumbar bone stress injuries occurred in players aged 17- and 18-years-old and were attributed to increases in bowling workload and intensity as the players progress towards more senior and elite cricket [24]. In addition, greater exposure to strength and conditioning training may result in increased muscle mass in these players which, in combination with an immature lumbar spine unable to withstand the demands placed upon it, could help explain the high incidence and prevalence of lumbar spine injuries seen within this study [24]. As such, management of bowling volumes, and ensuring adequate rest are strategies that may mitigate this risk in young, developing fast bowlers. The high rate of lumbar spine injuries in this cohort of players warrants future studies to explore the risk factors of this injury that can inform injury prevention strategies.

Another interesting finding from this study is the relatively low incidence of thigh injuries in comparison to other research in male cricket, where the hamstring or thigh region is consistently the most common injury location reported [[3], [4], [5]]. The low incidence of thigh injuries has also been seen in South African provincial age group cricketers, where 4.2 ​% of injuries were to the hamstring, and in First-Class Australian players where 10.5 ​% of injuries were to the thigh in <22-years-old compared to 19.4 ​% in 22–25-years-old, 25.1 ​% in 25–28-years-old, 26.6 ​% in 28–30-years-old and 18.3 ​% in >31-years-old [20,25]. A history of hamstring injuries and older age have been identified as strong risk factors in a number of sports, including cricket [26]. The younger age of the players in the present study could therefore explain the lower rate of hamstring injuries observed.

There is also a suggestion that a previous lumbar spine fracture may be a risk factor for a subsequent hamstring injury [27]. This has been shown in First-Class Australian cricketers whereby those with a previous lumbar spine stress fracture were 1.5 times more likely to sustain a subsequent hamstring injury, compared to those who have not sustained a lumbar spine stress fracture [28]. The associated hypertrophy from lumbar spine healing has been proposed to potentially lead to subsequent lumbar nerve root impingement which may increase the risk of subsequent lower limb muscle strains including hamstring injuries [27]. Therefore, it is possible that while at an increased risk of a bony injury, particularly to the lumbar spine region, this group of players are at a reduced risk of a hamstring muscle injury in comparison to older athletes due to their age. Conversely, younger cricketers with a lumbar spine stress fracture could be at an increased risk of sustaining a subsequent hamstring injury in the future, thus appropriate injury prevention strategies to mitigate this risk should be considered with these athletes. Future research is required, however, to provide further evidence to support this theory.

The inclusion of non-time loss and time-loss injuries in this study adds to the understanding of the injury risk to academy cricketers. There was a similar non-time loss and time-loss injury incidence reported, indicating that academy cricketers will continue to play and train whilst managing injuries that require medical attention. This has previously been seen in under-15 and under-17 schoolboy cricket [15]. While non-time loss injuries may not result in time away from cricket, they do have the potential to affect performance, as seen in international men's cricket [29] and increase demand on medical staff. Albeit in semi-professional male footballers, the risk of sustaining a time-loss injury within seven days of a minor or moderate non-time loss complaint has been reported as three to six times higher compared to the absence of any complaint [9]. Therefore, preventative measures and management strategies to minimise the severity of non-time loss injuries and minimise progression to time-loss injuries should also be considered in this group of athletes.

There was a decrease in annual non-time loss injury incidence in 2020/21 compared to the first two years and in 2021/22 compared to 2017/18. It is possible that the substantially reduced exposure to cricket in 2019/20 due to COVID-19 allowed for more effective management of non-time loss injuries through adequate rest and recovery. However, the access to medical staff for this cohort of players during this year is likely to have been limited due to the COVID-19 pandemic, therefore fewer non-time loss injuries may have been reported to medical staff and this is considered a limitation of this study.

### 4.1 Limitations

In this cohort of players, it was not possible to collect individual exposure time. Although this study conforms with the updated consensus statement for injury surveillance methods in cricket by reporting annual injuries/100 players/year [2], a limitation is the inability to report match injuries/1000 player days, thus potentially limiting the comparability of these results to other studies. Future studies in elite academy cricket should seek to report match injuries in line with the consensus statement. Unfortunately, it was not possible to include female academy players in this study as the same data was not available due to the restructuring of women's domestic cricket in England and Wales in 2021. Data pertaining to players role was not available, as such comparisons of injury incidence and prevalence by player role is lacking. The inability to breakdown injury by the age groups is a limitation of this study as injury profiles could differ in each age group. Another potential limitation of this study is the method of data entry for injuries. Injuries were recorded by academy physiotherapists or doctors and, as with any injury surveillance study, there is a risk for human error. This was mitigated by the lead author checking all injury data and consulting with the gatekeeper where necessary. To improve compliance, the ECB mandates standards for medical and injury records through annual science and medicine audits. Nonetheless, there is still a small risk of inaccurate injury records. The pressure to obtain a contract, particularly for older players in this cohort and the part-time nature of academies may have resulted in an under-reporting and non-reporting of injuries, the extent of which could not be determined in this study. Furthermore, injuries sustained by players particularly lumbar spine injuries in bowlers which have a prolonged injury prevalence may result in players not obtaining a contract for the next season. Both of these factors could potentially underestimate the extend of the injury problem in the elite male academy setting.

## 5 Conclusion

This study describes the injury risk in elite male academy players, with similar annual injury incidences observed over the four years. Overall, match bowling was the activity associated with the greatest risk of injury for players, having the highest injury incidence and prevalence, with lumbar spine injuries the most common body location and having the highest prevalence. Similar rates of time-loss and non-time loss injury incidence were observed, demonstrating the importance of establishing preventative measures for both of these injury types. The findings from this study provide robust evidence of the extent of the injury problem in male elite academy cricketers, which will allow future studies to identify risk factors and mechanisms of injury, and ultimately aid with the development of injury prevention strategies.

## Practical Implications

• The findings from the first analysis of elite male academy cricket injuries in England and Wales provides much needed evidence of the extent of the injury problem and appears to have a lower rate of injury compared to professional male cricket.
• Strategies that may help mitigate the risk of injury in young fast bowlers, specifically for lumbar spine injuries, should be implemented due to the high incidence and prevalence of injuries resulting from bowling.
• Injury prevention strategies should be developed specific for elite male academy players due to differences in injury profiles compared to professional male cricket players and target both time-loss and non-time loss injuries as similar rates were observed.

## Confirmation of ethical compliance

Ethical approval for this work was granted by the 10.13039/501100000835University of Bath Research Ethics Approval Committee for Health (REACH reference number: EP 20/21 065) and was carried out in accordance with The Code for Ethics of the Word Medical Association (Declaration of Helsinki) for experiments involving humans.

## Funding information

The research did not receive any specific grant form funding agencies in the public, commercial, or not-for-profit sectors. This study was undertaken as part of Amy Williams PhD Studentship joint funded by the 10.13039/501100000835University of Bath and ECB.

## Declaration of competing interest

Nicholas Peirce and Steve Griffin are employed by the England and Wales Cricket Board, the governing body for cricket in England and Wales. Ben Langley was employed by the England and Wales Cricket Board during the study years. Amy Williams PhD studentship is joint funded by the England and Wales Cricket Board and the ​University of Bath.
