# Supplementary material for: Annual incidence and prevalence of injuries in elite male academy cricketers: A 4-year prospective cohort study
Source: JSAMS Plus. 2023 Dec 26;3:100050. doi: 10.1016/j.jsampl.2023.100050 (PMC13008441; doi:10.1016/j.jsampl.2023.100050)
Supplement: Multimedia component 1 [file mmc1.docx]

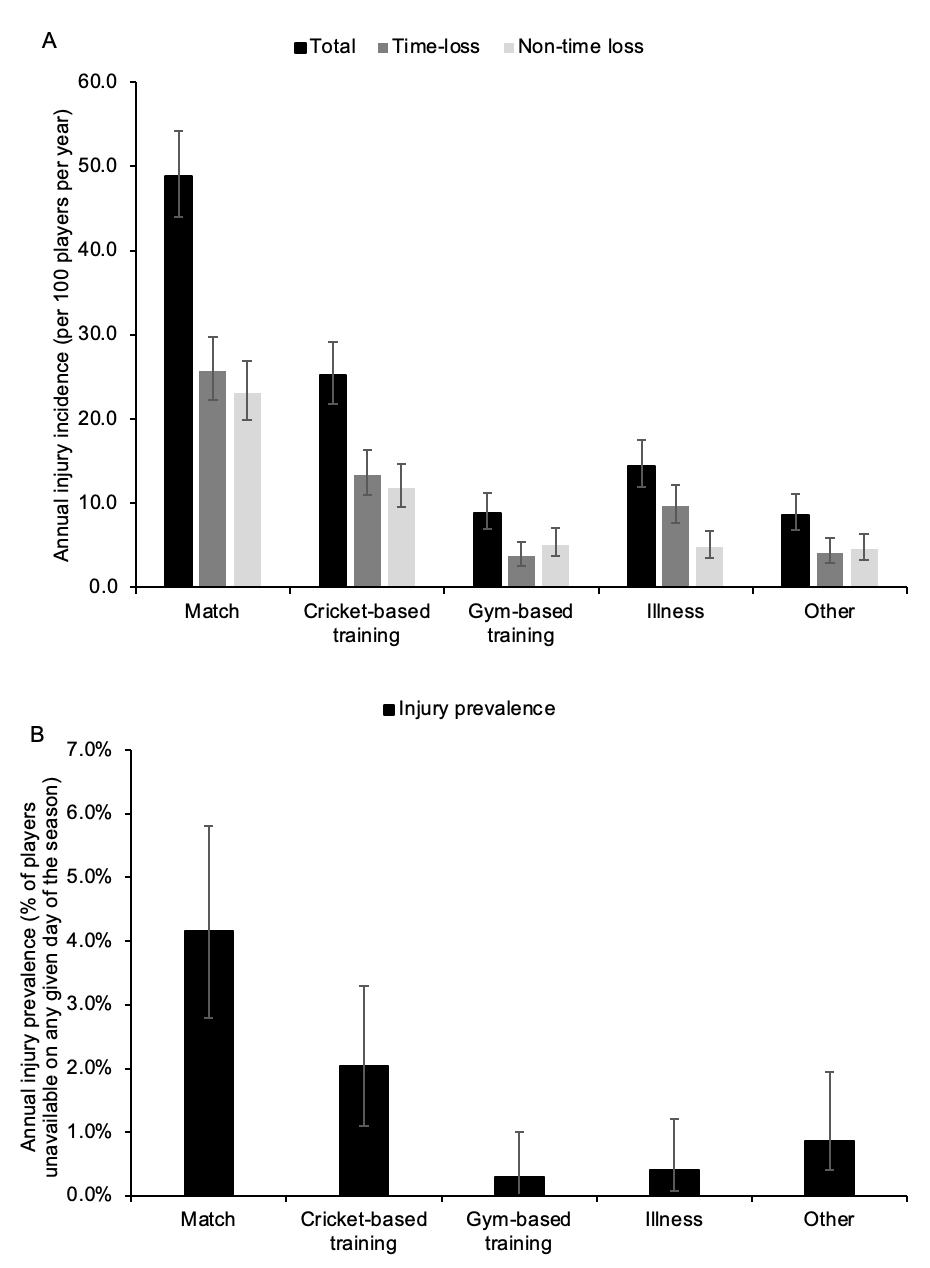


Figure S1. A) The average annual injury incidence (per 100 players per year) and B) the average annual injury prevalence (percentage of players unavailable on any given day of the year) by the problem type, with 95% CI error bars.
