# Supplementary material for: Annual incidence and prevalence of injuries in elite male academy cricketers: A 4-year prospective cohort study
Source: JSAMS Plus. 2023 Dec 26;3:100050. doi: 10.1016/j.jsampl.2023.100050 (PMC13008441; doi:10.1016/j.jsampl.2023.100050)
Supplement: Multimedia component 2 [file mmc2.docx]

Table S1. The average annual injury incidence (per 100 players per year) and prevalence (percentage of players unavailable on any given day of the year) by body location with 95% CI

| Body location | Injury incidence | | | Injury prevalence |
| --- | --- | --- | --- | --- |
|  | Total | Time-loss | Non-time loss |  |
| Lumbar spine | 15.3 (12.7, 18.4) | 8.4 (6.5, 10.8) | 6.9 (5.2, 9.1) | 2.9% (1.7%, 4.3%) |
| Hand | 12.1 (9.8, 14.9) | 6.3 (4.7, 8.4) | 5.8 (4.3, 7.8) | 0.6% (0.1%, 1.4%) |
| Ankle | 11.1 (9.0, 13.9) | 6.1 (4.5, 8.1) | 5.1 (3.7, 7.0) | 0.8% (0.3%, 1.8%) |
| Shoulder | 8.1 (6.3, 10.5) | 3.7 (2.5, 5.4) | 4.4 (3.1, 6.2) | 0.7% (0.2%, 1.6%) |
| Knee | 8.1 (6.3, 10.5) | 3.2 (2.1, 4.8) | 5.0 (3.6, 6.9) | 1.0% (0.4%, 2.0%) |
| Head | 7.0 (5.3, 9.2) | 3.2 (2.1, 4.8) | 3.9 (2.7, 5.6) | 0.2% (0.0%, 0.8%) |
| Thigh | 6.9 (5.2, 9.1) | 4.4 (3.1, 6.2) | 2.5 (1.6, 3.9) | 0.4% (0.1%, 1.2%) |
| Lower leg | 6.6 (5.0, 8.8) | 2.3 (1.5, 3.8) | 4.3 (3.0, 6.1) | 0.3% (0.0%, 1.0%) |
| Hip & groin | 6.3 (4.7, 8.4) | 2.9 (1.9, 4.4) | 3.4 (2.3, 5.1) | 0.3% (0.0%, 1.0%) |
| Abdomen | 5.8 (4.3, 7.8) | 4.1 (2.9, 5.9) | 1.7 (0.9, 2.9) | 0.4% (0.1%, 1.2%) |
| Foot | 4.7 (3.3, 6.5) | 2.9 (1.9, 4.4) | 1.8 (1.0, 3.1) | 0.4% (0.1%, 1.2%) |
| Thoracic spine | 2.1 (1.2, 3.4) | 0.8 (0.4, 1.8) | 1.2 (0.6, 2.4) | 0.0% (0.0%, 0.0%) |
| Elbow | 1.8 (1.0, 3.1) | 0.4 (0.1, 1.3) | 1.4 (0.7, 2.6) | 0.0% (0.0%, 0.0%) |
| Forearm | 1.2 (0.6, 2.4) | 0.1 (0.0, 1.0) | 1.1 (0.6, 2.2) | 0.0% (0.0%, 0.0%) |
| Chest | 1.1 (0.6, 2.2) | 0.7 (0.3, 1.7) | 0.4 (0.1, 1.3) | 0.3% (0.0%, 1.0%) |
| Wrist | 1.0 (0.5, 2.0) | 0.1 (0.0, 1.0) | 0.8 (0.4, 1.8) | 0.0% (0.0%, 0.0%) |
| Neck | 0.8 (0.4, 1.8) | 0.3 (0.1, 1.1) | 0.6 (0.2, 1.5) | 0.0% (0.0%, 0.0%) |
| Buttock & pelvis | 0.7 (0.3, 1.7) | 0.1 (0.0, 1.0) | 0.6 (0.2, 1.5) | 0.0% (0.0%, 0.0%) |
| Upper arm | 0.1 (0.0, 1.0) | 0.0 (0.0, 0.0) | 0.1 (0.0, 1.0) | 0.0% (0.0%, 0.0%) |
