# Supplementary material for: Annual incidence and prevalence of injuries in elite male academy cricketers: A 4-year prospective cohort study
Source: JSAMS Plus. 2023 Dec 26;3:100050. doi: 10.1016/j.jsampl.2023.100050 (PMC13008441; doi:10.1016/j.jsampl.2023.100050)
Supplement: Multimedia component 3 [file mmc3.docx]

Table S2. A breakdown of the illness type by percentage each year

| Medical illness | 2017/18 | 2018/19 | 2020/21 | 2021/22 |
| --- | --- | --- | --- | --- |
| Covid-19 | 0.0% | 0.0% | 79.5% | 52.2% |
| Gastrointestinal | 15.8% | 35.3% | 2.3% | 4.3% |
| Respiratory | 10.5% | 11.8% | 0.0% | 0.0% |
| Cardiovascular | 15.8% | 5.9% | 0.0% | 4.3% |
| Infection | 15.8% | 5.9% | 6.8% | 13.0% |
| Psychological | 10.5% | 0.0% | 0.0% | 0.0% |
| Other | 31.6% | 41.2% | 11.4% | 26.1% |
