# Supplementary material for: Annual incidence and prevalence of injuries in elite male academy cricketers: A 4-year prospective cohort study
Source: JSAMS Plus. 2023 Dec 26;3:100050. doi: 10.1016/j.jsampl.2023.100050 (PMC13008441; doi:10.1016/j.jsampl.2023.100050)
Supplement: Multimedia component 4 [file mmc4.docx]

Table S3. The average annual injury incidence (per 100 players per year) and prevalence (percentage of players unavailable on any given day of the year) by activity with 95% CI

| Activity | Injury incidence | | | Injury prevalence |
| --- | --- | --- | --- | --- |
|  | Total | Time-loss | Non-time loss |  |
| Match bowling | 17.7 (14.9, 21.1) | 10.3 (8.2, 12.9) | 7.4 (5.7, 9.7) | 2.1% (1.1%, 3.3%) |
| Match fielding | 11.3 (9.1, 14.0) | 5.2 (3.8, 7.2) | 6.1 (4.5, 8.1) | 0.7% (0.2%, 1.6%) |
| Training bowling | 10.6 (8.5, 13.2) | 5.5 (4.0, 7.5) | 5.1 (3.7, 7.0) | 1.2% (0.6%, 2.3%) |
| Gym-based training | 8.8 (6.9, 11.2) | 3.7 (2.5, 5.4) | 5.1 (3.7, 7.0) | 0.3% (0.0%, 1.0%) |
| Match batting | 7.4 (5.7, 9.7) | 3.6 (2.4, 5.3) | 3.9 (2.7, 5.6) | 0.5% (0.2%, 1.4%) |
| Training fielding | 7.4 (5.7, 9.7) | 3.2 (2.1, 4.8) | 4.3 (3.0, 6.1) | 0.4% (0.0%, 1.2%) |
| Training batting | 6.7 (5.1, 8.9) | 4.5 (3.2, 6.4) | 2.2 (1.3, 3.6) | 0.5% (0.1%, 1.4%) |
| Match wicket keeping | 1.0 (0.5, 2.0) | 0.7 (0.3, 1.7) | 0.3 (0.1, 1.1) | 0.0% (0.0%, 0.5%) |
| Training wicket keeping | 0.4 (0.1, 1.3) | 0.1 (0.0, 1.0) | 0.3 (0.1, 1.1) | 0.0% (0.0%, 0.5%) |
